# STAT3 modulates reprogramming efficiency of human somatic cells; insights from autosomal dominant Hyper IgE syndrome caused by STAT3 mutations

**DOI:** 10.1242/bio.052662

**Published:** 2020-09-10

**Authors:** Zhen Yu, Natalia I. Dmitrieva, Avram D. Walts, Hui Jin, Yangtengyu Liu, Xianfeng Ping, Elisa A. Ferrante, Lugui Qiu, Steven M. Holland, Alexandra F. Freeman, Guibin Chen, Manfred Boehm

**Affiliations:** 1 Translational Vascular Medicine Branch, National Heart, Lung, and Blood Institute, National Institutes of Health, Bethesda, MD 20892, USA; 2 Laboratory of Clinical Immunology and Microbiology, NIAID, NIH, Bethesda, MD 20892, USA

**Keywords:** Reprogramming, STAT3, Hyper IgE syndrome, iPSC

## Abstract

Human induced pluripotent stem cell (iPSC) technology has opened exciting opportunities for stem-cell-based therapy. However, its wide adoption is precluded by several challenges including low reprogramming efficiency and potential for malignant transformation. Better understanding of the molecular mechanisms of the changes that cells undergo during reprograming is needed to improve iPSCs generation efficiency and to increase confidence for their clinical use safety. Here, we find that dominant negative mutations in STAT3 in patients with autosomal-dominant hyper IgE (Job's) syndrome (AD-HIES) result in greatly reduced reprograming efficiency of primary skin fibroblasts derived from skin biopsies. Analysis of normal skin fibroblasts revealed upregulation and phosphorylation of endogenous signal transducer and activator of transcription 3 (STAT3) and its binding to the *NANOG* promoter following transduction with OKSM factors. This coincided with upregulation of NANOG and appearance of cells expressing pluripotency markers. Upregulation of NANOG and number of pluripotent cells were greatly reduced throughout the reprograming process of AD-HIES fibroblasts that was restored by over-expression of functional STAT3. NANOGP8, the human-specific NANOG retrogene that is often expressed in human cancers, was also induced during reprogramming, to very low but detectable levels, in a STAT3-dependent manner. Our study revealed the critical role of endogenous STAT3 in facilitating reprogramming of human somatic cells.

## Abstract (precis)

**Summary:** By using naturally-occurring mutations in a human disease, we demonstrate the critical role of endogenous STAT3 in modulating reprogramming efficiency in human somatic cells.

## INTRODUCTION

Pluripotent cells have the ability to generate all somatic lineages. *In vivo*, the property of pluripotency exists transiently in the inner cell mass (ICM) of the epiblast, a transient tissue that persist only for a few days. Isolation of cells at this stage and derivation of embryonic stem-cell (ESC) lines has made it possible to maintain pluripotency in culture indefinitely as long as they are maintained in a cell culture environment capable of inducing a transcriptional profile and epigenetic states resembling those of pluripotent epiblast cells (Hanna et al., 2010b; Nichols and Smith, 2012; Weinberger et al., 2016). Another source of pluripotent cell lines is the direct *in vitro* reprograming of somatic cells to pluripotency by ectopic expression of defined factors, yielding induced pluripotent stem cells (iPSCs) (Takahashi et al., 2007; Takahashi and Yamanaka, 2006).

Human iPSC technology has opened exciting opportunities for stem-cell-based therapies and has already been successfully used for applications such as *in vitro* disease modeling and drug screening (Inoue et al., 2014; Shi et al., 2017). However, despite great progress, several important issues remain to be addressed before this technology can be widely adopted for clinical use. These challenges include low reprograming efficiency, heterogeneity of iPSCs (mixture of cells at different states of pluripotency, Weinberger et al., 2016) with current protocols resulting in inefficient and inconsistent differentiation, and predisposition to mutations due to long-term culturing (Inoue et al., 2014; Shi et al., 2017). Better understanding of the molecular mechanisms of the changes that these cells undergo during reprograming is needed to improve the generation of homogeneous iPSC, mimicking pluripotent cells of preimplantation embryos that can be safely used in clinical practice (Koche et al., 2011; Polo et al., 2012; Takahashi and Yamanaka, 2016).

This study addresses the role of signal transducer and activator of transcription 3 (STAT3) in reprograming of human somatic cells into iPSC. In conjunction with core pluripotency transcription factors such as Oct4, Sox2 and NANOG, STAT3 occupies a central place in stem-cell signaling networks that regulate maintenance of pluripotency and self-renewal both *in vivo* and in ESCs and iPSCs cell lines *in vitro* (Nichols and Smith, 2012; Onishi and Zandstra, 2015). In the mouse embryo, STAT3 is highly expressed in oocytes and regulates the OCT4–NANOG circuitry necessary to maintain the pluripotent ICM, the source of *in vitro*-derived ESCs (Do et al., 2013). *In vitro*, maintenance of mouse ESC lines without the feeder layer of fibroblasts became possible when a strong activator of STAT3, leukemia inhibitory factor (LIF), was identified as the single factor that provides the ‘differentiation inhibitory activity’ originally produced by the feeder layer (Smith et al., 1988; Williams et al., 1988). Activation of STAT3 by LIF was found to be the driving mechanism and artificially-activated STAT3 could thus be used to sustain ESC self-renewal in the absence of LIF (Matsuda et al., 1999; Niwa et al., 1998; Raz et al., 1999). Further, inhibition of the simultaneously LIF activated MAPK/Erk pathway, which promotes differentiation, helped achieve more stable pluripotent states (Burdon et al., 1999, 2002). These findings identified STAT3 signaling as a major driving force for pluripotency maintenance and made it possible to culture ESC in defined serum-free medium with LIF and inhibitors of two kinases (Mek and GSK3) that promote differentiation, a condition known as 2i (Ying et al., 2008).

While LIF/STAT3 signaling has become a hallmark of pluripotency in rodent pluripotent stem cells, LIF has failed to support self-renewal of human ES cells derived from blastocysts (Dahéron et al., 2004; Thomson et al., 1998) as well as human iPSCs obtained by direct *in vitro* reprogramming (Takahashi et al., 2007; Takahashi and Yamanaka, 2016). In current protocols, the self-renewal capability of human pluripotent cells in culture is dependent on fibroblast growth factor 2 (FGF2) and transforming growth factor-β/avidin signaling (Vallier et al., 2005), requiring the presence of factors modulating these signaling pathways in the culturing environment. The molecular mechanisms underlying these differences are not completely understood. Reprograming that follows the expression of OSKM factors involves a series of chromatin remodeling events with the ultimate activation of endogenous factors that drive pluripotency (Koche et al., 2011), many of which are downstream transcriptional targets of STAT3 (Chen et al., 2008; Tang et al., 2012). In this study, we have revisited the question of the role of STAT3 in human cell reprograming. To test whether endogenous STAT3 could mediate and facilitate the reprograming of human cells, we used STAT3-deficient primary skin fibroblasts derived from patients with autosomal-dominant hyper IgE (Job's) syndrome (AD-HIES). AD-HIES is a primary immunodeficiency caused by dominant negative mutations in STAT3 (Holland et al., 2007; Minegishi et al., 2007). Several dozen heterozygous mutations in the *STAT3* gene that result in AD-HIES have been identified (Villarino et al., 2017; Vogel et al., 2015). These mutations are located primarily in the DNA-binding or the protein-dimerization (SH2) domains resulting in a 1:1 mixture of wild-type and mutated proteins, which allows for a residual normal function of about 20–30% STAT3 dimers composed of wild-type protein molecules (Vogel et al., 2015). Patients with both mutation types have very similar clinical presentation, suggesting that they induce similar functional deficiencies on STAT3 protein.

Here, we demonstrate that a deficiency in endogenous STAT3 in cells from AD-HIES patients greatly reduces reprograming efficiency of human somatic cells into iPSC generated with a widely used protocol using lentiviral transduction of OSKM factors and E8 media (Chen et al., 2011). This decreased derivation efficiency was accompanied by decreased upregulation of NANOG in cell cultures undergoing reprogramming, a key event in the transcriptional network reorganization during reprograming to pluripotency (Jopling et al., 2011; Saunders et al., 2013; Takahashi and Yamanaka, 2016). Our analysis revealed that endogenous STAT3 binds to the promoter of the *NANOG* gene during the reprograming process coinciding with its increased expression, suggesting that STAT3 might directly contribute to this upregulation. Although to a much lower extent than regular NANOG, expression of the human-specific NANOGP8 retrogene, often expressed in human cancers (Jeter et al., 2009; Jeter et al., 2011; Zhang et al., 2013, 2006), was also slightly induced by the reprograming process in a STAT3-dependent manner. The data reveal critical contributions of endogenous STAT3 to cellular remodeling of human somatic cells into pluripotent states after forced introduction of OKSM factors.

## RESULTS

### Reduced reprogramming efficiency of iPSCs from skin fibroblasts of AD-HIES patients harboring loss-of-function mutation in *STAT3*

**Fig. 1.**
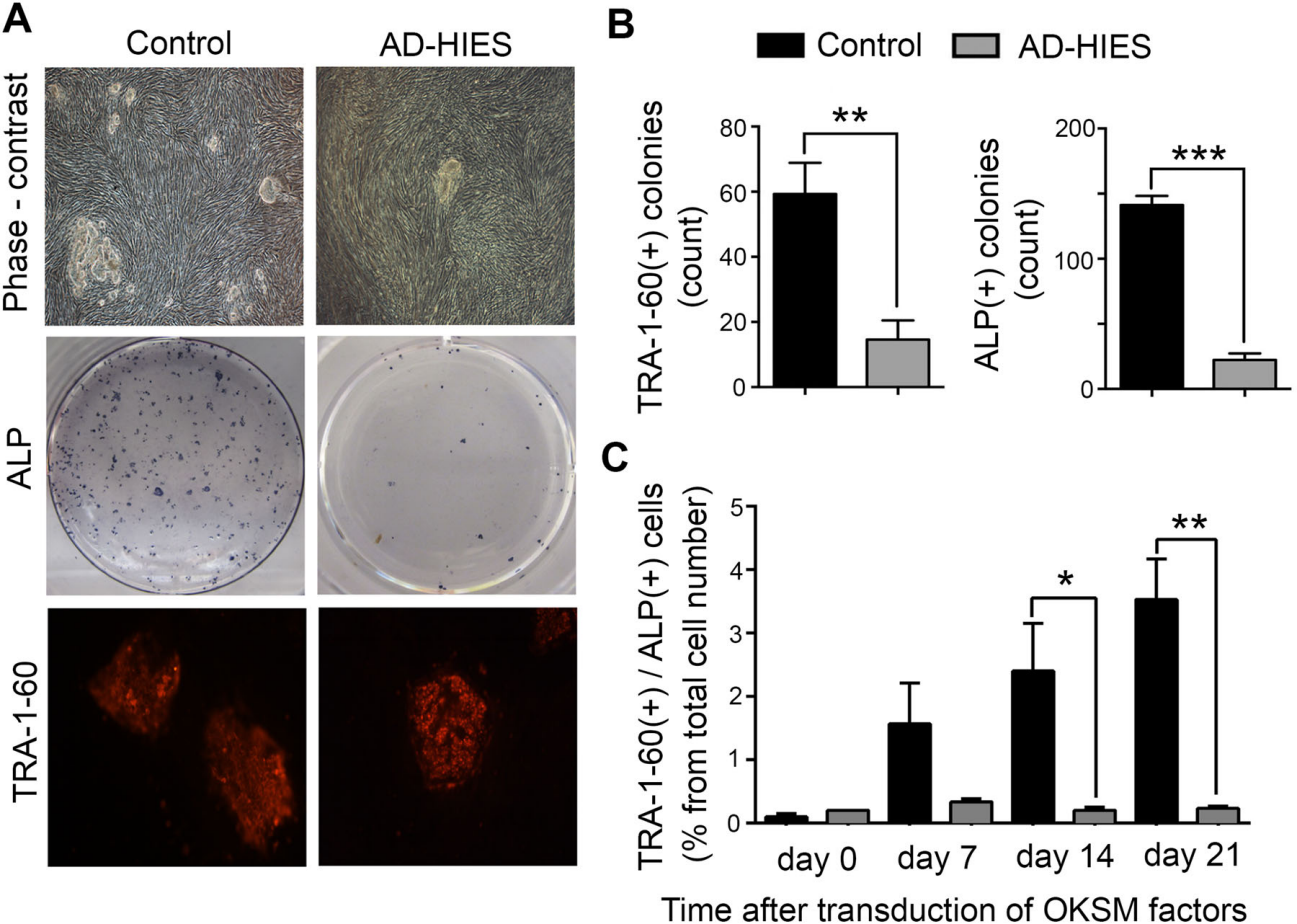
**Reduced reprogramming efficiency of AD-HIES skin fibroblasts to iPSCs.** Skin fibroblasts generated from seven AD-HIES patients and from four healthy volunteers (control) were subjected to the reprogramming procedure using lentiviral delivery of four transcription factors: human OCT4, KLF4, SOX2 and cMYC (OKSM) and appearance of pluripotent cells and colonies was analyzed. ALP and TRA1-60 were used as markers of pluripotency. See the Materials and Methods for more details. (A,B) At the end of 21 days of reprograming, the number of pluripotent colonies obtained from AD-HIES fibroblasts is greatly reduced as compared to control fibroblasts. (A) Representative images of pluripotent colonies. Top panels*:* phase contrast images. Middle panels: staining for ALP activity. Positive colonies are blue dots. Bottom panels: immunocytochemical staining for TRA1-60 (red). (B) Quantification of TRA1-60 and ALP positive colonies. Data are presented as the number of colonies per well of a six-well plate (mean±s.e.m., control *n*=4, AD-HIES *n*=7, **P<0.01, ***P<0.001, two-tailed unpaired *t*-test). (C) Time course of proportion of pluripotent cells throughout the reprogramming procedure. Data are presented as the percentage of double positive for ALP and TRA1-60 cells at indicated time points, analyzed by flow cytometry (mean±s.e.m., *n*=3, *P<0.05, **P<0.01, two-tailed unpaired *t*-test). See Table S1 for information about patient samples used in these experiments.

To test whether endogenous STAT3 plays a role in the remodeling of human somatic cells to pluripotency, we generated primary skin fibroblasts derived from patients with AD-HIES harboring dominant negative mutations in STAT3 (Table 1). We employed a reprogramming procedure using lentiviral delivery of four transcription factors: human OCT4, KLF4, SOX2, and cMYC (OKSM) (Chen et al., 2011) and observed greatly reduced reprograming efficiency of primary human fibroblasts derived from skin biopsies of AD-HIES patients compared to those from healthy control volunteers (Fig. 1). By reprogramming day 21, significantly less pluripotent colonies had developed from AD-HIES fibroblasts compared to control fibroblasts, as assessed visually from the characteristic morphology of the colonies and by staining for pluripotency markers such as alkaline phosphatase (ALP) activity and TRA-1-60 (Fig. 1A,B). The analysis of finally formed iPSCs showed that, despite lower reprogramming efficiency, they expressed pluripotency markers and differentiated into three germ layers similar to control iPSCs (Jin et al., 2019). Therefore, we proceeded with more detailed analysis of the reprogramming time course.

**Table 1.**
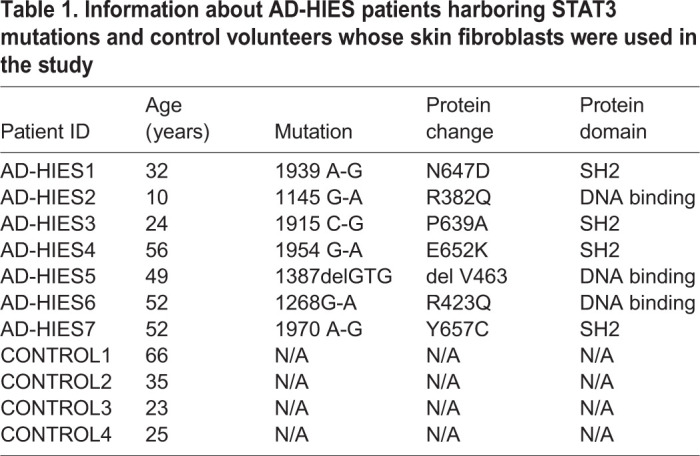
**Information about AD-HIES patients harboring STAT3 mutations and control volunteers whose skin fibroblasts were used in the study**

The reprogramming from somatic cells to iPSC is a stochastic process with only a minor fraction of cells expressing OKSM giving rise to iPSC colonies. It involves waves of chromatin remodeling that result in a major shift of expression profiles that affect small fractions of cells and ultimately resembles expression patterns of ESCs to then develop pluripotent colonies (Koche et al., 2011; Polo et al., 2012). In order to clarify the timing of events in the reprogramming process, we performed a time course analysis of the appearance of pluripotent cells after OKSM transduction (Fig. 1C) in AD-HIES and control cells. The number of pluripotent cells, double positive for ALP and TRA-1-60, gradually increased in transduced control fibroblasts reaching approximately 4% by day 21. By comparison, AD-HIES cells were significantly less successful: the trend to decreased number of cells expressing pluripotency markers is evident as early as day 7 with no further increase in the percentage of pluripotent cells (Fig. 1C). These results indicate that this deficiency occurs at the beginning of the reprograming process, likely affecting the initial chromatin reorganization and the expression of endogenous pluripotency drivers*.*

### STAT3 dependence of reprogramming from human skin fibroblasts to iPSCs

**Fig. 2.**
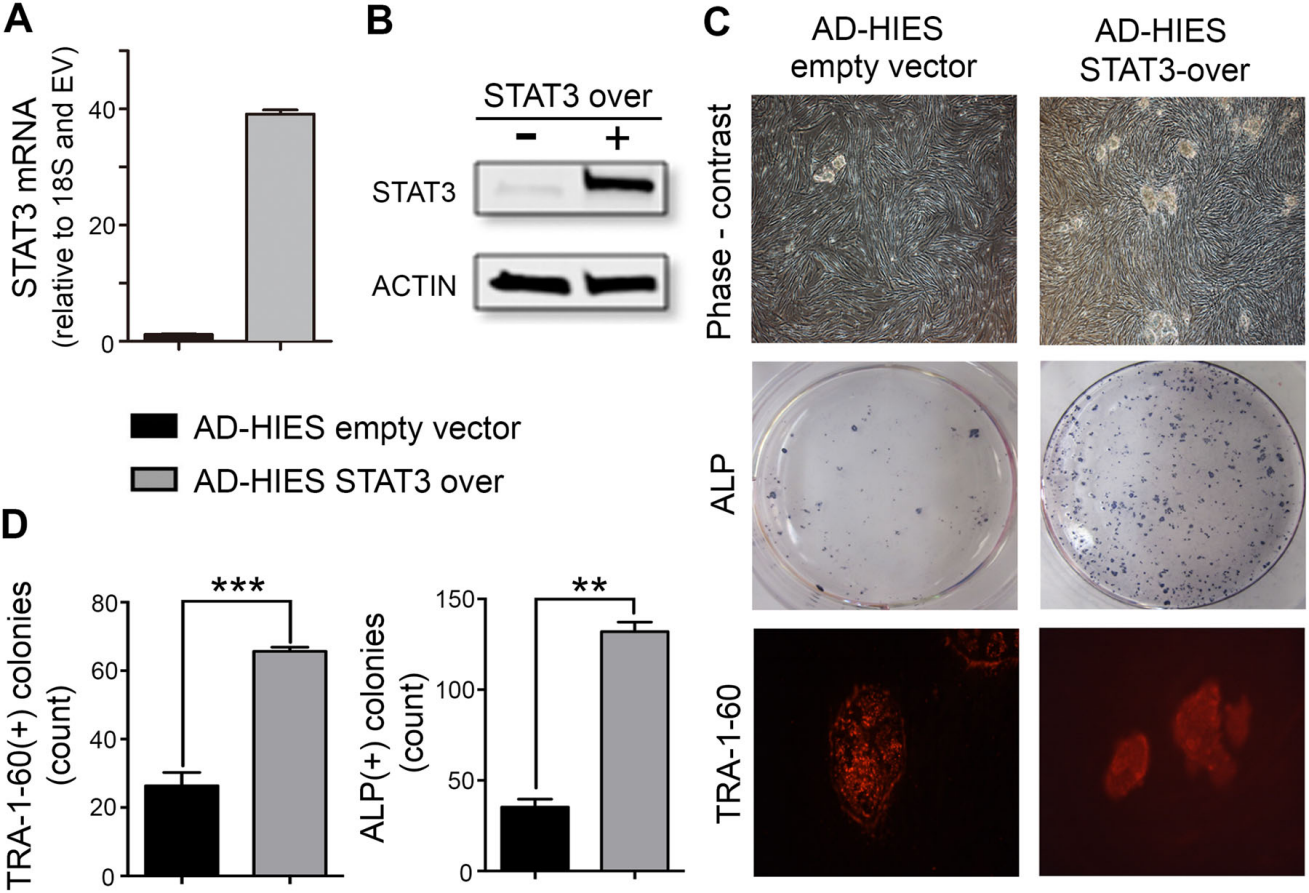
**Overexpression of STAT3 restores reprogramming efficiency in AD-HIES skin fibroblasts.** Fibroblasts from AD-HIES patients were transduced via lentiviral delivery with STAT3 cDNA (AD-HIES-STAT3 over) or empty vector (AD-HIES empty vector, EV), subjected to the reprogramming procedure and appearance of pluripotent colonies was analyzed as in Fig. 1. (A,B) Verification of STAT3 overexpression in AD-HIES skin fibroblasts (A) transduction increased STAT3 mRNA. Quantification was done by RT-PCR and data are presented relative to empty vector values (B) transduction increased STAT3 protein. Representative western blot. (C,D) Overexpression of wild-type STAT3 protein increased the number of pluripotent colonies formed at the end of 21 days in the reprogramming procedure. (C) Representative images of pluripotent colonies. Top panels: phase contrast images; middle panels: staining for ALP activity, positive colonies are blue dots; bottom panels: immunocytochemical staining for TRA1-60 (red). (D) Quantification of TRA1-60 and ALP positive colonies. Data are presented as the number of colonies per well of a six-well plate (mean±s.e.m., *n*=3, **P<0.01, ***P<0.001, two-tailed unpaired *t*-test). See Table S1 for information about patient samples used in these experiments.

**Fig. 3.**
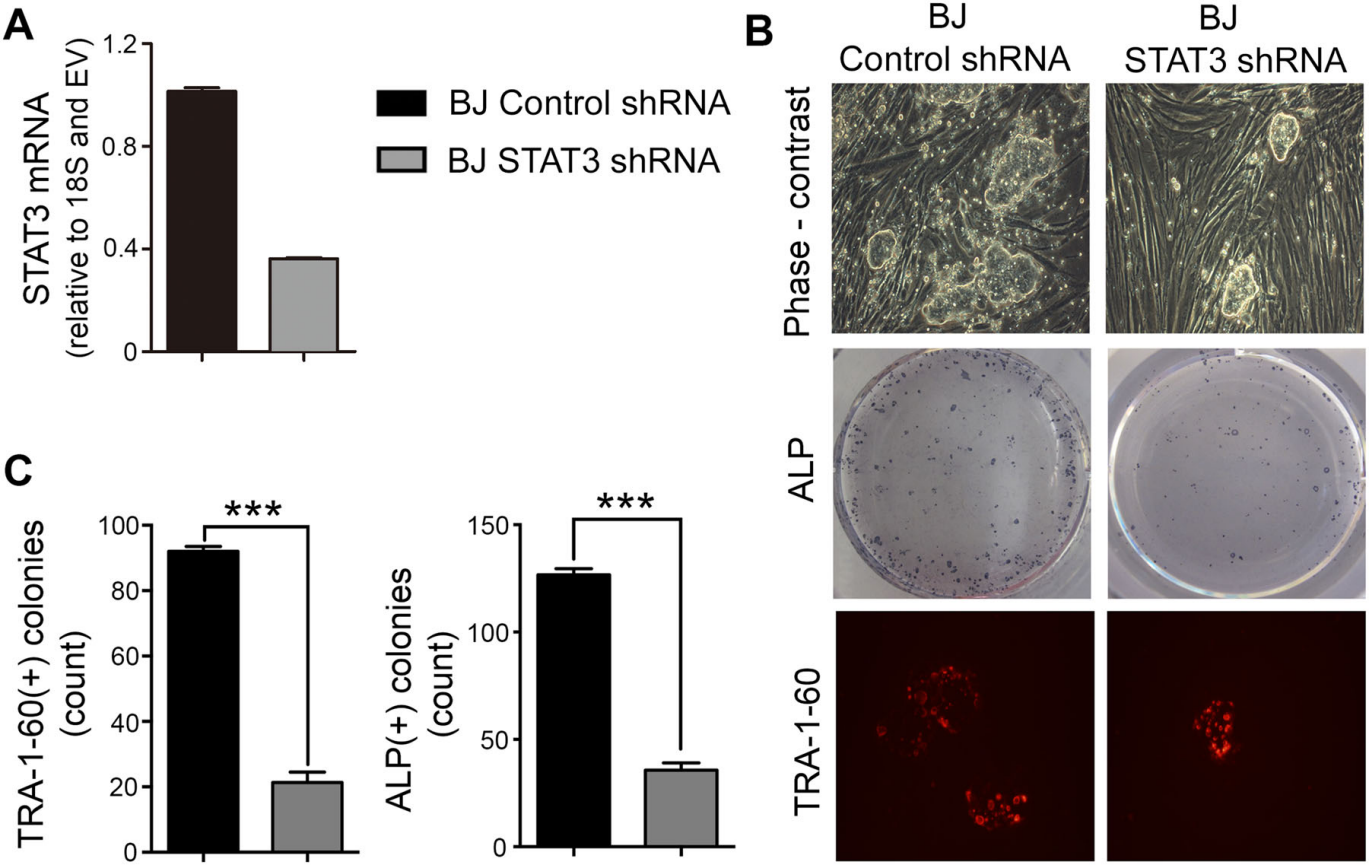
**Knockdown of STAT3 decreases reprogramming efficiency in human skin fibroblasts (BJ cell line).** STAT3 shRNA was delivered into healthy control skin fibroblasts (BJ cell line) through lentiviral vector. Non-silencing control shRNA was used as control. The fibroblasts were subjected to the reprogramming procedure and pluripotent colonies were analyzed as in Fig. 1. (A) Knockdown verification. STAT3 mRNA was decreased by shRNA. Quantification was done by RT-PCR and data are presented relative to empty vector. (B,C) Knockdown of STAT3 decreased the number of pluripotent colonies formed at the end of 21 days in the reprogramming procedure. (B) Representative images of pluripotent colonies. Top panels: phase contrast images; middle panels: staining for ALP activity, positive colonies are blue dots. Bottom panels: immunocytochemical staining for TRA1-60 (red). (C) Quantification of TRA1-60 and ALP positive colonies. Data are presented as the number of colonies per well of a six-well plate (mean±s.e.m., *n*=3, ***P<0.001, two-tailed unpaired *t*-test).

To validate the functional implication of STAT3 in the reprogramming defects observed in AD-HIES cells, we investigated whether overexpression of functional wild-type STAT3 could improve reprogramming efficiency of AD-HIES fibroblasts (Fig. 2) and whether knocking down STAT3 in normal skin fibroblasts could mimic the reprogramming defects (Fig. 3). Lentiviral delivery of wild-type *STAT3* elevated expression of both STAT3 mRNA (Fig. 2A) and protein (Fig. 2B) and improved reprogramming efficiency of AD-HIES fibroblasts, evident in the increased number of pluripotent colonies positive for TRA1-60 and ALP (Fig. 2C,D) by day 21 of reprogramming procedure. On the other hand, knockdown of *STAT3* in BJ normal skin fibroblasts cell line (No. CRL-2522, ATCC) by STAT3 shRNA (Fig. 3A) decreased the number of pluripotent colonies formed by reprogramming day 21 as compared to control shRNA (Fig. 3B–D).

These results confirm that the decreased reprogramming efficiency of AD-HIES skin fibroblasts results from the reduced function of STAT3 mediated by a disease-causing genetic mutation in the *STAT3* gene. Our results further highlight the importance of endogenous STAT3 for the successful reprogramming of human skin fibroblasts to iPSC when overexpression of OKSM transcription factors is used as a reprogramming approach.

### STAT3 expression and phosphorylation is increased during reprogramming, coinciding with upregulation of NANOG

**Fig. 4.**
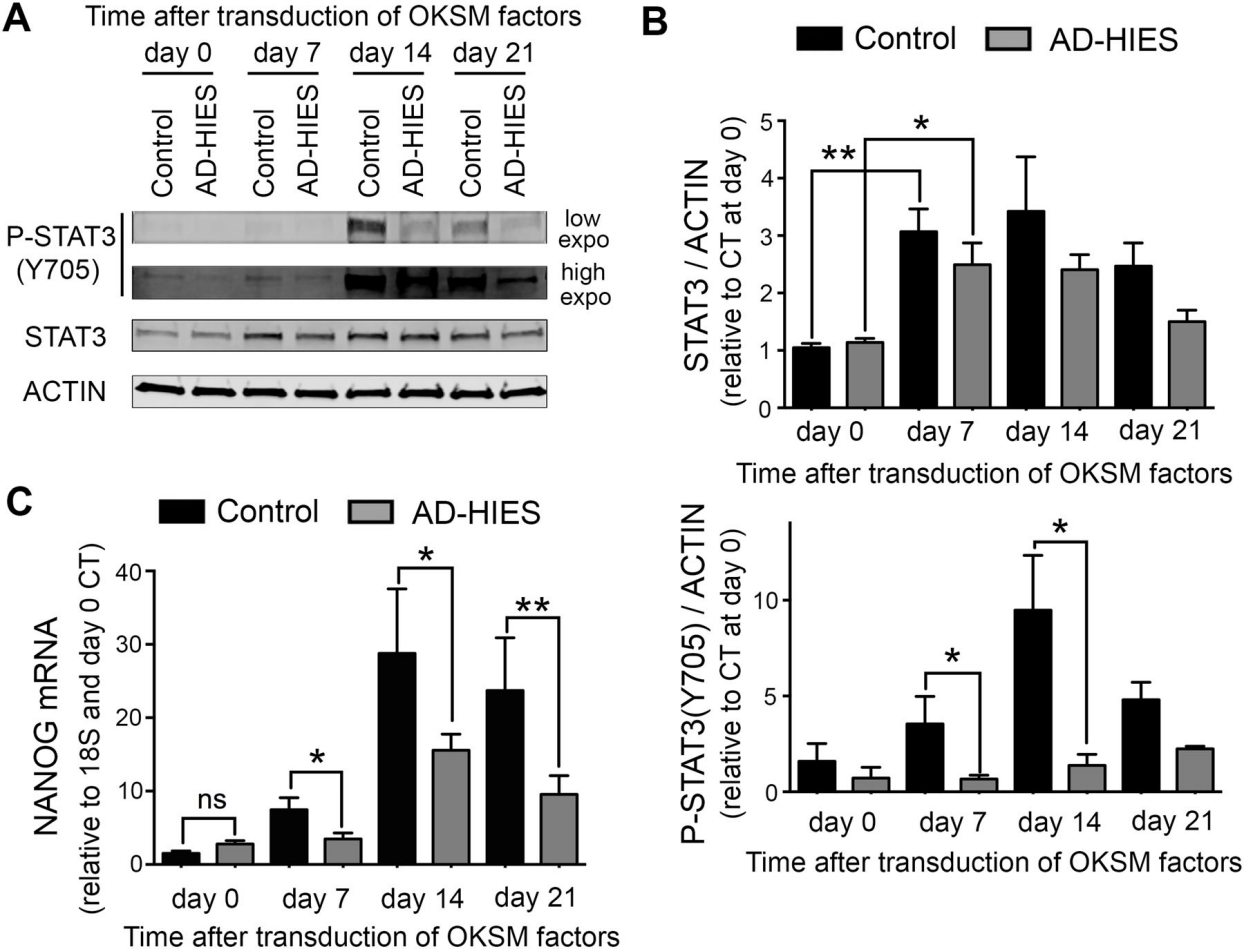
**STAT3 is upregulated and phosphorylated during reprogramming with time course coinciding with STAT3-dependent increase in expression of NANOG.** Skin fibroblasts generated from AD-HIES patients and from healthy volunteers (control) were subjected to the reprogramming procedure as described in Fig. 1. STAT3 and NANOG levels were analyzed during reprogramming at indicated time points. (A,B) Western blot analysis of STAT3 protein expression and phosphorylation at tyrosine 705 associated with its transcriptional activation. (A) Representative western blot images of p-STAT3 Y705 and total STAT3. (B) Western blot quantification by densitometry, upper panel: expression of total STAT3 protein increases by day 7 and remains elevated to a similar degree in both control and AD-HIES cells; lower panel: the level of P-STAT3 Y705 gradually increases in control cells reaching a maximum at day 14 of reprogramming. P-STAT3 Y705 is greatly reduced in AD-HIES cells. Data are presented relative to STAT3/β-ACTIN of control at d0 (mean±s.e.m., *n*=3, *P<0.05, **P<0.01, two-tailed unpaired *t*-test). (C) NANOG mRNA expression gradually increases reaching a maximum at day 14. The level of NANOG mRNA is reduced in AD-HIES cells. Quantification was performed by RT-PCR (mean±s.e.m., control: *n*=4, AD-HIES, *n*=7, *P<0.05, two-tailed unpaired *t*-test). See Table S1 for information about patient samples used in these experiments.

Having determined that reprograming of skin fibroblasts to iPSC is STAT3-dependent, we next analyzed how STAT3 protein level and activity changes during reprogramming. Phosphorylation of STAT3 at Tyrosine 705 is required for activation of its transcriptional activity (Zhong et al., 1994). Western blot analysis demonstrated that expression level of total STAT3 protein is increased by reprogramming day 7 and remains elevated through day 21 (Fig. 4A,B, upper panel). The level of phosphorylated STAT3 follows the same time course, reaching a maximum at day 14 and decreasing by day 21 (Fig. 4A,B, lower panel). In AD-HIES cells, STAT3 expression is similarly increased but the level of phosphorylated protein is greatly reduced, suggesting that the AD-HIES STAT3 mutation does not affect expression levels but rather prevents its normal phosphorylation and activity during reprograming (Fig. 4A,B). This points to the existence of a positive feedback loop initiated by activated STAT3 for this phosphorylation process during reprograming. It is worth noting that the actual decrease in AD-HIES STAT3 transcriptional activity is even higher than would be expected from a decreased amount of phosphorylated protein, since the AD-HIES mutations do not affect the phosphorylation site but prevent STAT3 from binding to its DNA target sites.

During the reprograming process, changes in molecular events following OKSM transcription-factor overexpression lead to activation of endogenous pluripotency genes encoding OCT4, NANOG and SOX2 important for establishment and maintenance of the pluripotent state independent of the transgenes (Hanna et al., 2010b). NANOG upregulation is a key event in the transcriptional network reorganization that occurs during reprograming to pluripotency (Jopling et al., 2011; Saunders et al., 2013; Takahashi and Yamanaka, 2016). In mouse ESCs and iPSC, STAT3 stimulates and maintains NANOG expression upon treatment with LIF through direct binding to its specific binding sites within the *NANOG* gene promoter, as shown by chromatin immunoprecipitation (ChIP) (Chen et al., 2008; Do et al., 2013) and increased activity of luciferase reporter containing NANOG-promoter sequences (Suzuki et al., 2006). In order to see how deficiency in endogenous STAT3 affects NANOG upregulation during reprograming of human cells, we analyzed the expression of NANOG in relation to STAT3 expression and phosphorylation. This analysis demonstrated that NANOG mRNA expression increases in control cells as early as day 7 and continues to increase throughout the reprograming procedure reaching maximum level at day 14 (Fig. 4C) resembling the time course of STAT3 protein expression and phosphorylation. Induction of NANOG mRNA expression in AD-HIES cells was greatly attenuated, which is consistent with STAT3 dependence of NANOG reactivation during reprograming (Fig. 4C).

### Preferential STAT3-dependent increase in NANOG expression as compared to NANOGP8 during reprogramming of human skin fibroblasts

**Fig. 5.**
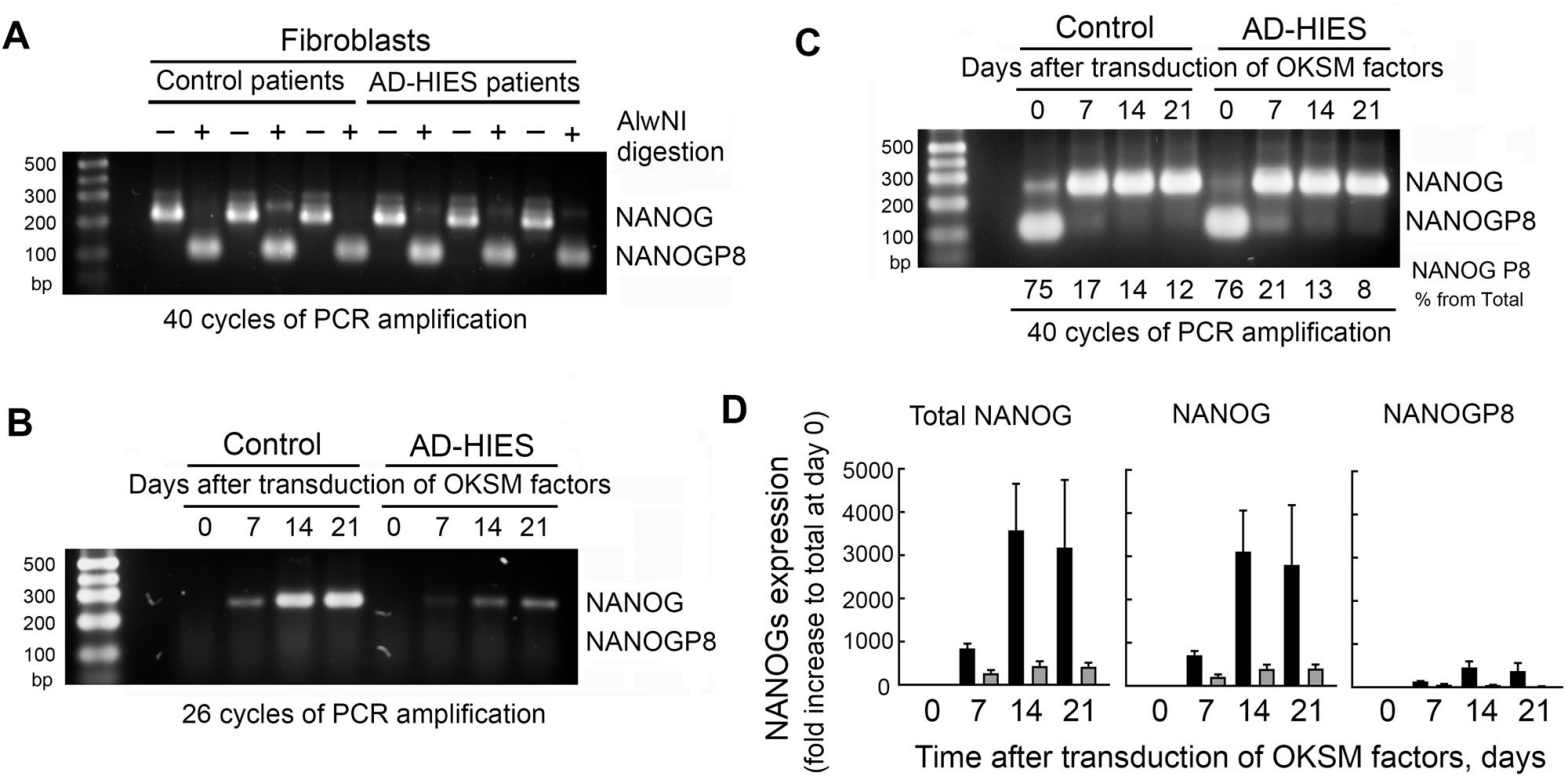
**NANOG, not its pseudogene NANOGP8, is upregulated in a STAT3-dependent manner during reprogramming of human skin fibroblasts to iPSCs.** Control and AD-HIES skin fibroblasts were subjected to the reprogramming protocol as described in Materials and Methods and analyzed at indicated time points. (A–D) Analysis of relative expression of NANOG and NANOGP8. Total NANOG RT-PCR products were digested with AlwNI restriction endonuclease that specifically cuts only NANOGP8 and fragments were analyzed by agarose gel electrophoresis (see Materials and Methods for details). Three control and three AD-HIES cell lines were analyzed for all experiments. (A) NANOGP8 is the predominant form of NANOG in both control and AD-HIES skin fibroblasts. The cDNA region containing AlwNI site in NANOGP8 was amplified for 40 cycles. (B) NANOG is the predominant form that is upregulated during reprogramming. PCR amplification was stopped during logarithmic phase to reflect relative expression level. (C,D) NANOG expression is increased during reprograming both in control and AD-HIES cells but to a much smaller extent in AD-HIES. NANOGP8 expression also increased but overall levels are much lower. (C) 40 cycles of PCR amplification were performed and % of NANOGP8 in total NANOG was determined by densitometry of corresponding bands, (D) quantification of NANOG and NANOGP8 expression during reprogramming based on qPCR quantification of total NANOG (left panel) and proportion of NANOG P8 obtained from Fig. 5C (mean±s.e.m., *n*=3). See Table S1 for information about patient samples used in these experiments.

Analysis of NANOG in human cells is complicated by the presence of ten very similar NANOG pseudogenes. One of them, NANOGP8, encodes a full length protein that differs by only 2–3 amino-acid changes (Booth and Holland, 2004), making it indistinguishable when analyzed by western blot or by regular qPCR as in Fig. 4C (Zhang et al., 2006). In addition to being a key regulator of pluripotency, NANOG has been described as a crucial transcription factor in various types of cancer. Several studies investigating which NANOGs were expressed in cancer cells and tissues identified that NANOGP8 was the most prevalent NANOG expressed in many human cancers and contributed to their ‘stemness’ and proliferative capacity (Jeter et al., 2009, 2011; Zhang et al., 2013, 2006). Moreover, NANOGP8 is as active as NANOG in the reprogramming process of both human and murine fibroblasts into induced pluripotent stem cells (Palla et al., 2014). With this in mind, we analyzed the relative contribution of regular NANOG and NANOGP8 in the STAT3-dependent changes modulating the expression of total NANOG during our reprogramming of skin fibroblasts into iPSCs. We used previously published approaches to distinguish NANOG and NANOGP8 mRNA based on the digestion of RT-PCR products with restriction endonuclease AlwNI, an enzyme that identifies a palindromic hexanucleotide sequence present in NANOGP8 but not in NANOG at position 144 relative to the translational start site (Zhang et al., 2013). PCR amplification of cDNA fragments containing this site and digestion of the PCR products with AlwNI showed that low levels of NANOG expressed in both control and AD-HIES skin fibroblasts (Fig. 4C) is predominantly due to NANOGP8 (Fig. 5A), whereas the increase in the total level of NANOG during reprogramming is mostly due to an increase in the expression of regular NANOG (Fig. 5B). Since NANOGP8 expression level was much lower, it was not detected on gel after 26–28 PCR cycles, while NANOG amplification was still in logarithmic phase in comparison (Fig. 5B). To further quantify changes in the level of NANOGP8, we amplified cDNA for 40 cycles and estimated the relative proportion of NANOG and NANOGP8 in the total NANOG by densitometry of their corresponding bands (Fig. 5C). We then used these data to recalculate expression levels of NANOG and NANOGP8 based on the qPCR quantification of total NANOG expression (Fig. 5D, left panel) and relative proportions of NANOG and NANOGP8 (Fig. 5C). This analysis showed that both NANOG and NANOGP8 increased during reprogramming but NANOG was the highly predominant form (Fig. 5D). Increases of both NANOG and NANOGP8 were attenuated in AD-HIES consistent with STAT3 dependence of this regulation (Fig. 5B,D).

### Preferential binding of STAT3 to the promoter of *NANOG* as compared to *NANOGP8* gene during reprogramming of human skin fibroblasts

**Fig. 6.**
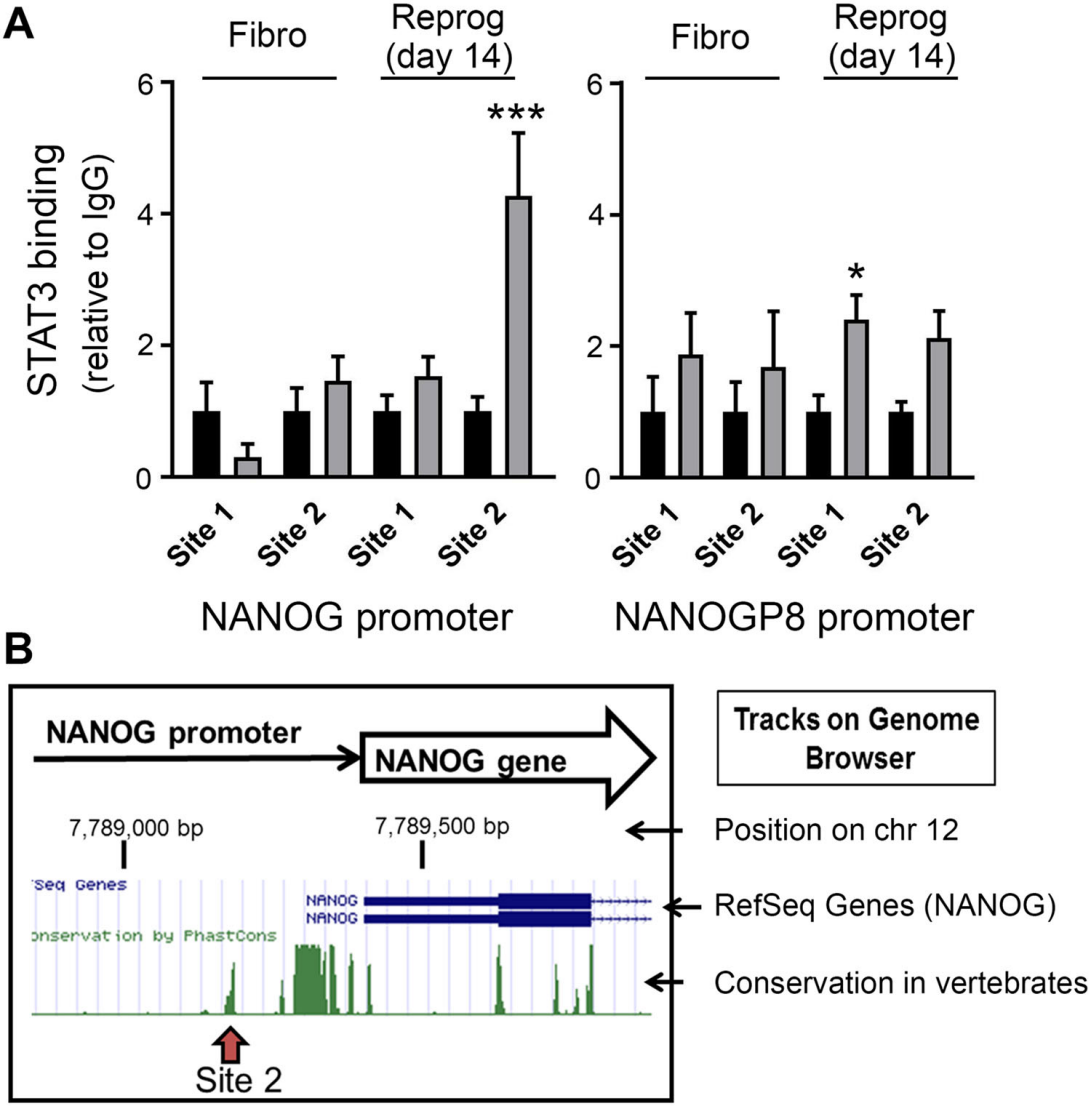
**STAT3 binds to NANOG promoter during reprogramming of human skin fibroblasts to iPSCs but not in fibroblasts.** Binding of STAT3 to promoters of NANOG and NANOGP8 was analyzed by ChIP. Samples for ChIP were collected from control skin fibroblasts and at day 14 of reprogramming. (A) Analysis of STAT3 binding to potential binding sites that contain STAT3 binding sequences in NANOG and NANOGP8 promoters. ChIP, see Materials and Methods for details, data are plotted as mean±s.e.m., *P<0.05, ***P<0.001, two-tailed *t*-test relative to IgG, *n*=3. (B) Location of the binding Site 2 in NANOG promoter relative to the transcription start site of NANOG gene (RefSeq Genes track of the UCSC Genome Browser). The site overlaps with highly conserved regions on the ‘Conservation in vertebrates’ track of the browser. See also Fig. S2 for the binding sites locations and Table S2 for primers sequences. See Table S1 for information about patient samples used in these experiments.

Having found that NANOG upregulation during reprograming through overexpression of OKSM transcriptional factors is modulated by STAT3, we next tested whether STAT3 directly binds to the promoters of NANOG and NANOGP8 (Fig. 6). We performed this analysis at reprogramming day 14 because levels of *P*-STAT3 (Y705) (Fig. 4A,B) and NANOG mRNA expression (Fig. 4C) reach a maximum by this time and the number of pluripotent cells increases (Fig. 1C), indicating ongoing active reorganization of chromatin structures and gene expression profile. Analysis of promoter sequences of *NANOG* and *NANOGP8* genes showed that they both have potential STAT3 binding sites (see Materials and Methods for more details). We designed two primer sets for each promoter covering the regions containing the STAT3 binding sites. Locations of these regions are shown on Fig. S2. ChIP analysis showed that STAT3 does not bind *NANOG* or *NANOGP8* promoters in fibroblasts but binds the region spanning binding site 2 in *NANOG* promoter during reprograming (Fig. 6). Slight enrichment in STAT3 binding to the promoter of *NANOGP8* was also detected (Fig. 6A) but to a much lower extent than STAT3 binding to site 2 of the promoter of regular *NANOG,* consistent with predominant upregulation of regular NANOG during reprogramming. The STAT3 binding site 2 is located in a highly conserved region of the NANOG promoter (Fig. 6B), consistent with its important regulatory role. In summary, activation and binding of STAT3 to the *NANOG* promoter during reprogramming and attenuated upregulation of NANOG expression in AD-HIES fibroblasts in combination with decreased reprogramming efficiency of AD-HIES fibroblasts to iPSC suggest that upregulation of NANOG during reprograming through overexpression of OKSM factors in human skin fibroblasts is regulated by endogenous STAT3.

## DISCUSSION

The data presented here support the role of endogenous STAT3 in the reprograming of human somatic cells into iPSCs. In mouse cells, STAT3, together with core pluripotency transcription factors such as Oct4, Sox2 and NANOG, occupies a central place in stem-cell-signaling networks regulating the maintenance of pluripotency and self-renewal both *in vivo* and in ESCs and iPSCs cell lines *in vitro* (Nichols and Smith, 2012; Onishi and Zandstra, 2015). The importance of STAT3 signaling in mouse pluripotency is highlighted by the fact that its activator LIF is a necessary component of cell culture media that have been developed for stable pluripotency maintenance as well as for reprograming (Matsuda et al., 1999; Niwa et al., 1998; Raz et al., 1999; Williams et al., 1988). During reprogramming of mouse cells, exogenous stimulation of STAT3 signaling increases efficiency of the transition to ground state pluripotency (Yang et al., 2010) and enables the induction and stabilization of a naïve pluripotent state (van Oosten et al., 2012). However, the role of STAT3 in human pluripotency is not as clear and LIF in cell culture media is not able to maintain pluripotency of either human embryonic cells or iPSCs (Dahéron et al., 2004; Takahashi and Yamanaka, 2016; Thomson et al., 1998; Vallier et al., 2005).

The difference in the STAT3 role for pluripotency regulation in mouse and human cells was initially attributed to differences in genetic background and it was concluded that STAT3 was not needed for maintenance of pluripotency and modulation of STAT3 signaling was not a promising target for method improvement in human iPSC derivation and maintenance. However, analysis of transcriptional and epigenetic profiles have revealed that these differences could be explained by the different states of pluripotency that mouse and human ESCs/iPSCs acquire in cell culture, which are stabilized *in vitro* by different growth conditions (Hanna et al., 2010b; Weinberger et al., 2016). It has since been shown that the pluripotent state of human ESCs/iPSCs in culture conditions corresponds to that of the mouse-derived epiblast stem cells (EpiSC), designated as ‘primed’ pluripotent state as opposed to ‘naïve’ or ‘ground’ state of mouse ESC derived from ICM (Nichols and Smith, 2012). The primed state is prone to differentiation whereas the naïve ESCs correspond to a more immature state of pluripotency of preimplantation embryo ICM that is stabilized in culture by stimulation of FGF2/avidin signaling rather than LIF/STAT3 similar to human cells.

Following these discoveries, the importance of STAT3 signaling for human pluripotency was re-established when it was demonstrated that exposure of EpiSC-like pluripotent human cells, including human ESC and human iPSCs, to LIF/STAT3 is able to revert them to a ground state pluripotency. Similar to the mouse ESC, this conversion can be boosted by cultivating cells in 2i conditions (2i: GSK3b inhibitor and ERK1/2 inhibitor) (Hanna et al., 2010a,b) in combination with other inhibitors of differentiation promoting signaling (Chan et al., 2013; Chen et al., 2015; Gafni et al., 2013; Pastor et al., 2016; Theunissen et al., 2014). These findings are helping to reconcile the differences between mouse and human cells and suggest that the STAT3 role in establishing pluripotency and its maintenance might be more similar between species than was initially assumed.

**Fig. 7.**
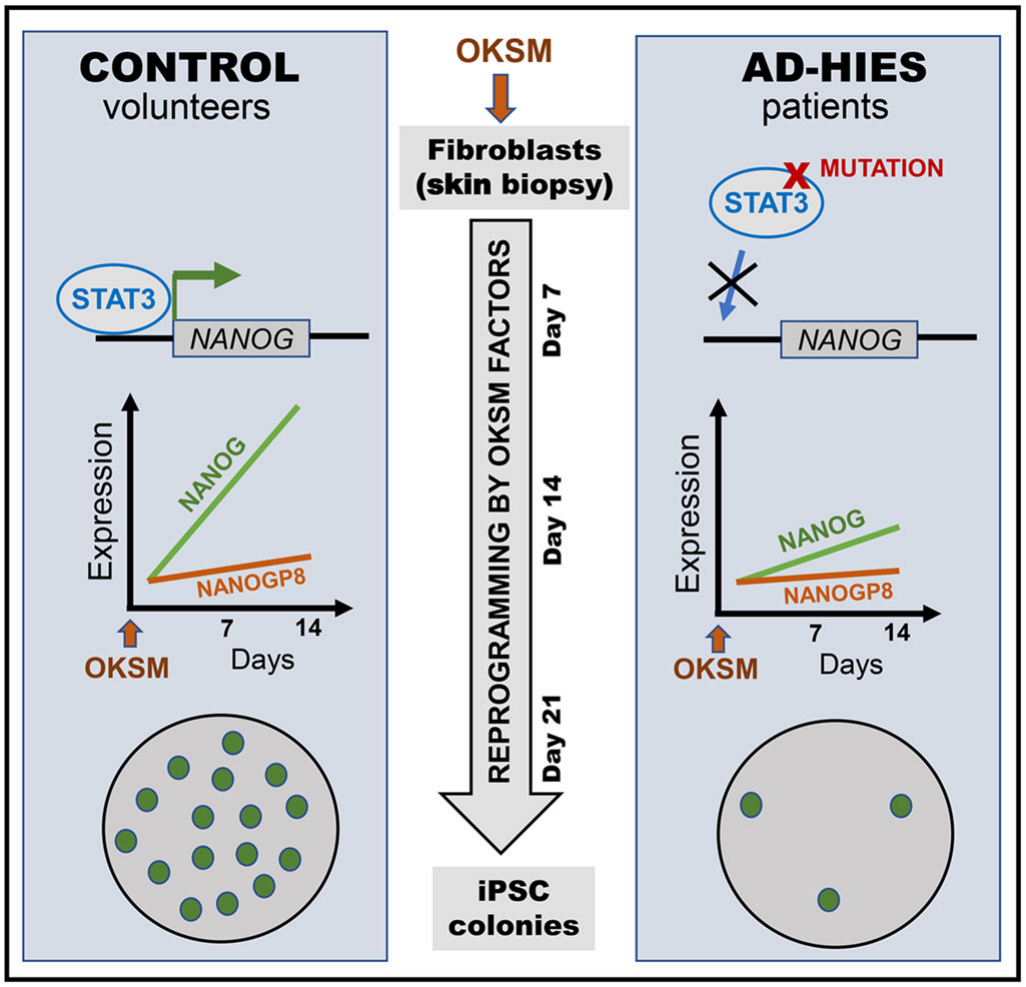
**Overview of the study findings.** Human fibroblast transduction with OKSM factors results in STAT3 activation and binding to the *NANOG* promoter with upregulation of NANOG and a small elevation of its retrogene NANOGP8, ultimately leading to iPSC colony formation. Consistent with STAT3 dependence, NANOG expression and pluripotent colony numbers are greatly reduced throughout the reprograming process of fibroblasts derived from AD-HIES patients harboring STAT3 mutations.

In this study, we show that normal function of endogenous STAT3 is needed for efficient reprograming of human somatic cells into iPSC (Fig. 7). This conclusion is made based on greatly reduced reprograming efficiency of primary skin fibroblasts derived from patients with AD-HIES syndrome, carrying dominant negative mutations in STAT3 (Fig. 1). The STAT3 dependence of this reprograming efficiency was confirmed by its improvement following overexpression of functional wild-type STAT3 in AD-HIES fibroblasts (Fig. 2) and by recapitulating reprogramming deficiency by knocking-down STAT3 in normal skin fibroblasts (Fig. 3). Further analysis demonstrated that during reprograming, as STAT3 protein expression is increased, it is activated, as evidenced by its phosphorylation (Fig. 4A,B), and it binds to its transcriptional binding site within the NANOG promoter (Fig. 6). These events coincide with increasing NANOG expression levels (Fig. 5) and the appearance of cells expressing pluripotency markers (Fig. 1C). In AD-HIES cells with reduced STAT3 function, all these events are attenuated, accompanied by greatly reduced numbers of successfully reprogrammed pluripotent cells. These results reveal the critical role of endogenous STAT3 in facilitating reprogramming of human somatic cells.

Analysis of NANOG is complicated by the presence of a fully functional pseudogene, NANOGP8, encoding a full-length protein that differs by only 2–3 amino-acid changes (Booth and Holland, 2004) and cannot be distinguished by regular western blot or PCR (Zhang et al., 2006). NANOGP8 is expressed in many cancers (Jeter et al., 2009, 2011; Zhang et al., 2013) and its ability to substitute for NANOG in reprograming activity (Palla et al., 2014), prompted us to analyze the relative contribution of NANOG and NANOGP8 in STAT3-dependent upregulation of total NANOG during our reprogramming procedure (Figs 5 and 6). The analysis demonstrated that STAT3 predominantly binds to the NANOG promoter and NANOG is the predominantly upregulated form during reprograming. However, NANOGP8 was also detectable in primary skin fibroblasts and was induced by the reprograming procedure, indicating that its promoter becomes more accessible for upregulation. NANOGP8 is a human-specific retrogene and it has been proposed that its expression in cancers could explain higher the predisposition to cancers in humans than other primates (Fairbanks et al., 2012). The findings suggest that testing iPSCs and their derivatives for NANOGP8 expression could be beneficial to decrease probability of malignant transformations.

In conclusion, our study demonstrates that normal function of endogenous STAT3 is critical for reprograming of human somatic cells into iPSCs initiated by lentiviral transduction of OSKM factors and performed in the absence of exogenous stimulation of STAT3 signaling. These findings, together with studies showing ability of LIF/STAT3 stimulation to revert EpiSC-like ‘primed’ pluripotent human cells to ground state pluripotency (Chan et al., 2013; Chen et al., 2015; Gafni et al., 2013; Hanna et al., 2010a,b; Pastor et al., 2016; Theunissen et al., 2014), support the important role of STAT3 during both the establishment and the maintenance of induced pluripotency in human cells.

The findings of this study point to endogenous STAT3 signaling being an important regulator of reprogramming of human somatic cells to iPSC. Due to its functions as a hub protein for multiple cellular signaling pathways and as a transcription factor with multiple transcriptional targets, STAT3 serves as a key regulator of multiple cellular processes such as cell survival, cell proliferation, migration, metabolism and chromatin remodeling (Demaria et al., 2014; Hirano et al., 2000; Wingelhofer et al., 2018; Yu et al., 2014). Many of these processes are involved in the series of transformations that cells undergo during the reprograming process, such as chromatin opening, increased proliferation rate, metabolic changes and acquisition of resistance to apoptosis and senescence (David and Polo, 2014; Gaspar-Maia et al., 2011). Further studies on which of these processes are affected by STAT3 deficiency could provide new insights into molecular mechanisms of reprograming and may help discover new approaches for increasing reprograming efficiency of human somatic cells to iPSC.

## MATERIALS AND METHODS

### Human subjects

Study subjects were evaluated under a National Institute of Allergy and Infectious Diseases (NIAID) Institutional Review Board-approved natural history of HIES protocol at the Clinical Center at the National Institutes of Health (NIH). Study subjects were diagnosed with AD-HIES using a diagnostic scoring system comprising of immunological and non-immunological features (Woellner et al., 2010). The diagnosis was confirmed by the identification of *STAT3* mutations listed in Table 1.

### Derivation of patient-specific skin fibroblasts

Four control and seven AD-HIES patient-derived fibroblasts lines were generated from 3–4 mm punch skin biopsies following informed consent under protocols approved by NHLBI IRB. The skin biopsy sample was further cut into 1 mm pieces and digested for 1 h at 37C in 10 ml of 0.1% Collagenase Type II (No.17101-015, Thermo Fisher Scientific)/0.25 U ml^−1^ Dispase (No. 17105-0411, Thermo Fisher Scientific)/PBS solution. The pieces were then transferred to two wells of a six-well culture plate, covered with cover slips to facilitate attachment and cultured in Dulbecco's modified Eagle medium (DMEM) supplemented with 20% fetal bovine serum (FBS) and antibiotics in a 20% O_2_, 5% CO_2_ incubator. Fibroblast outgrown from the explants were passaged after 3–4 weeks when they occupied most of the well's surface. The fibroblasts were then cultured in DMEM medium supplemented with 10% FBS (No. S10250, Atlanta biological, Flowery Branch, GA, USA) and antibiotics.

### Reprogramming of skin fibroblasts into iPSCs

IPSCs were generated from control and AD-HIES skin fibroblasts by lentiviral delivery of four transcription factors: human OCT4, KLF4, SOX2, and cMYC (OKSM) as previously described (Beers et al., 2012; Chen et al., 2011; Jin et al., 2016, 2019). Briefly, the fibroblasts were seeded in six-well plate at a density of 2×10^5^ per well. After 24 h, the cells were transduced with the Human STEMCCA Cre-Excisable Constitutive Polycistronic (OKSM) Lentivirus reprogramming kit (No.SCR545, EMD Millipore, Darmstadt, Germany) (Sommer et al., 2009). Cells were harvested 3–4 days after transduction and re-plated on six-well plates coated with Matrigel (no. 354230, Corning, USA). On the following day, E7 medium without TGF-β and supplemented with 1 μM hydrocortisone and 100 μM butyrate was added to cells and replaced every other day. After 2 weeks of transduction, cells were changed to full E8 medium (Stemcell Technology, Vancouver, Canada). iPSC colonies were collected 21 days post-transduction, maintained in full E8 medium and passaged with 0.5 mM EDTA as previously described (Beers et al., 2012).

### STAT3 overexpression

Full-length *STAT3* cDNA was purchased from Dharmacon (#7727). Using Invitrogen's Gateway Cloning System, the *STAT3* cDNA was subcloned to pLenti6.3⁄V5-DEST (Invitrogen, V53306). The virus was produced in HEK293FT cells using the ViraPower™ HiPerform™ Lentiviral Gateway Expression Kit (Invitrogen, K5330-00). Fibroblasts were virally transduced for 24–36 h and screened for puromycin resistance to identify stably transfected cells.

### shRNA knockdown

STAT3 was knocked down with a human ‘GIPZ lentiviral shRNA’ viral particle purchased from Dharmacon (RHS4531-NM_003150) including STAT3 shRNA or non-silencing control shRNA viral particles. Normal human skin fibroblasts (BJ) (no. CRL-2522, ATCC, Manassas, VA, USA) were transduced with for 24–36 h and screened for puromycin resistance for stable transfection.

### Quantification of pluripotent colonies by staining with pluripotency markers

#### TRA-1-60 surface marker expression analysis

Live cells were directly stained using GloLIVE Human Pluripotent Stem Cell Live Cell Imaging Kit (no. SC023, R&D). Anti-hTRA-1-60 antibodies were added directly to the cells for 30 min, washed with cell culture medium and imaged with Olympus IX71 microscope. Positive colonies in each well of six-well plates were counted manually.

#### ALP staining

Staining was performed with a SIGMAFAST BCIP/NBT kit (Sigma-Aldrich) by following the manufacturer's instructions.

### Quantification of TRA-1-60 and ALP double-positive cells by flow cytometry

The cells were digested to a single cell suspension at different time points after transduction by incubation with Trypsin-EDTA (0.25%, 25200056, Gibco) for 1 min. The cells were stained with mouse anti-human Alkaline Phosphatase-Alexa Fluor 488 (no. 56149, BD Pharmingen) and anti-human-TRA1-60-PE (no. 330610, Biolegend, San Diego, CA, USA). Analysis was performed on a BD FACSCanto Flow Cytometer (BD Biosciences, San Jose, CA, USA) and the results were analyzed using FlowJo software (FlowJo, LLC).

### RNA extraction and quantification by real-time PCR

Total RNA was extracted from cultured cells using the RNeasy Mini Kit (no. 74134, QIAGEN, Valencia, CA, USA). The RNA was converted to cDNA by reverse transcription using TaqMan Reverse Transcription Reagents (N8080234; Applied Biosystems). mRNA levels were measured by real-time PCR using iQ SYBR Green Supermix (Bio-Rad) on an MJ Research Dyad Disciple thermal cycler with Chromo 4 fluorescence detector (Bio-Rad). The specificity of the amplified PCR products was confirmed by analysis of the melting curves. The primers used for qPCR are shown in the Table S3. Quantification was performed by comparative CT method and 18S ribosomal RNA was used as an endogenous control. The relative copy number of a target was calculated for each sample [2 ^– (Ct( target mRNA) – Ct (18S rRNA)^] and normalized to the copy number in the corresponding control sample (specified in the figure legends).

### Western blot

Western blot analysis was performed by generating immunoblots of proteins separated by SDS-PAGE. All cells on the plate were bulk lysed in RIPA buffer supplemented with protease and phosphatase inhibitors. Primary antibodies against p-STAT3 (Tyr705) (9145; Cell Signaling Technology), STAT3 (9139; Cell Signaling Technology), β-Actin (3700, Cell Signaling Technology) were used in conjunction with anti-rabbit-IRDye800CW (no. 926-32211, Li-Cor, Lincoln, NE, USA) and anti-mouse-IRDye680RD (no. 926-68070, Li-Cor) as secondary antibodies. Immunoblots were scanned and integral fluorescence (IF) from each band was measured using Odyssey Infrared Imaging System (Li-COR Biosciences, Lincoln, NE, USA).

### Analysis of relative proportion of NANOG and NANOGP8 in mRNA expression of Total NANOG measured by qPCR

Total RNA was extracted with RNeasy Plus Mini Kit (no. 74134, Qiagen, Valencia, CA, USA). As NANOGP8 is an intronless retrogene, it is not possible to avoid amplification of NANOGP8 from genomic DNA by designing primers spanning introns. In order to ensure removal of all genomic DNA, we performed on column treatment with DNase and the absence of NANOG amplification was tested on the extracted RNA (Fig. S1). RNA (2 µg) was converted to cDNA by reverse transcription using a high-capacity cDNA RT Kit (no. 4368814, Applied Biosystems). Total NANOG mRNA levels were quantified by real-time PCR using QuantiFast SYBR Green PCR Kit (no. 204054, Qiagen, Valencia, CA,USA). Quantification was performed by comparative CT method and 18S ribosomal RNA was used as an endogenous control. The relative copy number of total NANOG mRNA was calculated for each sample as 2 ^– (Ct( NANOG) – Ct (18S rRNA)^.

PCR products digested with AlwNI according to the manufacturer's protocol (New England Biolabs, Beverly, MA, USA) were purified with the QIAquick PCR Purification Kit (no. 28104, Qiagen) and analyzed by electrophoresis on a 3% (w/v) agarose gel.

### ChIP

ChIP was performed using the Enzymatic Chromatin IP kit (no. 9003, Cell Signaling Technology, Danvers, MA, USA). Briefly, cells were crosslinked with 1% formaldehyde for 10 min. Chromatin was digested with MNase to generate fragments from 150 bp to 900 bp. For each sample, chromatin from one confluent six-well plate was immunoprecipitated with 10 µg of anti-Stat3 antibody (no. sc-13035, Santa Cruz Biotechnology, Dallas, TX, USA) or with normal rabbit IgG (No.2729, Cell Signaling Technology, Danvers, MA, USA). The protein in the samples was enzymatically digested to further purify the DNA. The number of DNA fragments containing target sequences in input chromatin and in chromatin immunoprecipitated (IP) with anti-STAT3 and IgG were quantified with a QuantiFast SYBR Green PCR Kit (no. 204054, Qiagen). Four target sequences were quantified, two containing the STAT3 binding sites in the NANOG promoter and two containing Stat3 binding sites in the NANOGP8 promoter (see ‘Primer design for Stat3 ChIP’ section for the target sequences). Quantification was performed by comparative CT method. The relative to input DNA copy number of each target sequence for each IP sample was calculated as 2 ^– (Ct( IP DNA) – Ct (Input DNA))^. The number of copies of each target sequence in Stat3 ChIP was normalized by the copy number of IgG ChIP.

### Primer design for Stat3 ChIP

Four primer pairs for specific regions containing STAT3 binding sites close to the transcription start site of the NANOG and NANOG P8 genes were designed using the Primer-BLAST tool (http://www.ncbi.nlm.nih.gov/tools/primer-blast/). The regions of the NANOG and NANOG P8 genes that were tested by ChIP are shown on Fig. S2 and the primer sequences are listed in Table S3. The primers’ specificities were verified by analysis of the melting curves of the PCR products obtained at the end of SYBR Green qPCR reaction. Each produced a single peak.

### Statistical analysis

Statistical analyses were done using GraphPad Prism7 software. All values are shown as mean±s.e.m. *P*-values were calculated with a two-tailed Student's *t*-test, and *P*<0.05 (*) was considered significant.

## Supplementary Material

Supplementary information
