# Supplementary material for: STAT3 modulates reprogramming efficiency of human somatic cells; insights from autosomal dominant Hyper IgE syndrome caused by STAT3 mutations
Source: Biol Open. 2020 Sep 10;9(9):bio052662. doi: 10.1242/bio.052662 (PMC7502598; doi:10.1242/bio.052662)
Supplement: Supplementary information [file biolopen-9-052662-s1.pdf]

Supplementary Information

Fig. S1 (related to Fig. 5). Quality check for the absence of genomic DNA contamination in RNA extracted for analysis of expression levels of NANOG and NANOGP8 shown on Fig 5. Genomic DNA contamination interferes with the quantification of the expression of NANOG gene because of the existence of an intronless retrogene, NANOGP8. Total RNA was extracted with *RNeasy Plus Mini Kit* (QIAGEN) containing a genomic DNA elimination column. Additional on-column treatment with DNase was performed to ensure complete removal of genomic DNA. The RNA was converted to cDNA. PCR amplification of NANOG was performed for 40 cycles using 4 random RNA samples and one cDNA sample. Absence of amplification from RNA samples confirms the absence of contamination with genomic DNA.

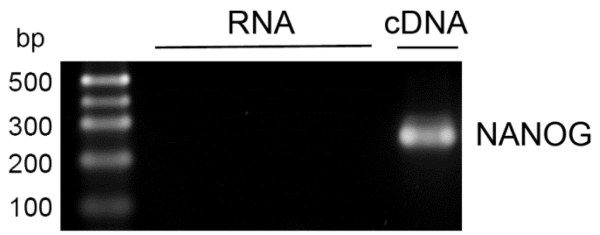

Biology Open • Supplementary information

Fig. S2 (related to Fig. 6). Location of primers targeting potential Stat3 binding sites in promoters of A) NANOG and B) NANOGP8 genes. 2000bp promoter sequences were retrieved using the Ensembl genome browser and searching for the presence of potential Stat3 binding sites (TT(N)$_{4\text{-}6}$AA). Multiple sites were found in both promoters and 2 primer pairs were designed for each promoter, targeting regions containing clusters of potential sites. Promoter sequences were aligned to human genome using the Blat tool from the UCSC Genome Browser (Dec. 2013 (GRCh38/hg38) Assembly). The location of sites targeted by the primers are shown relative to the transcription start sites of NANOG **(A)** and NANOGP8 **(B)** genes shown on RefSeq Genes track of the browser. “Conservation in vertebrates” and “DNase I Hypersensitivity” tracks show potential regulatory sites in the promoters. Site 2 in the NANOG promoter is located in a highly conserved region. **C)** Representative example of chromatin fragmentation results obtained in the Stat3ChIP experiments shown on Fig. 6. Predominant fragment size is one nucleosome (150 bp) but fragments up to 900bp are also present indicating that binding to sites located withing 900bp distance from targeted sequences would be detected too, although with less sensitivity.

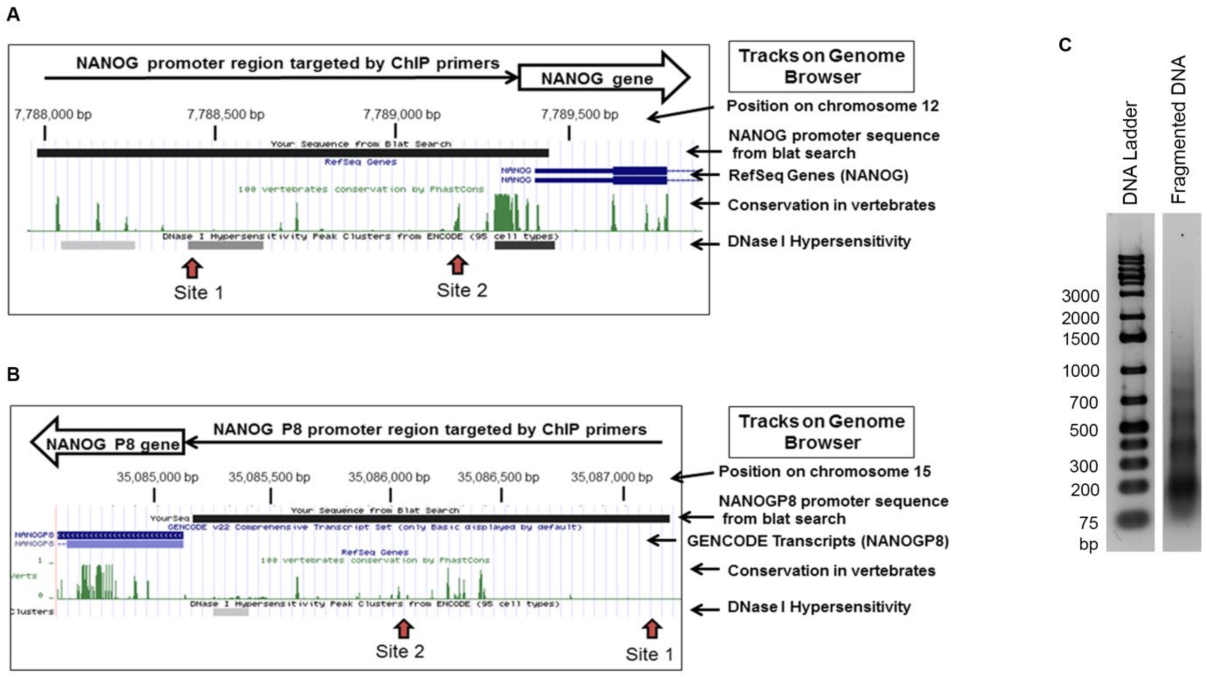

Biology Open • Supplementary information

Table S1. List of patients' and controls' cells that were used in experiments of the study.

See Table 1 for information about the patients and STAT3 mutations.
Controls and patients are listed as: Patient ID (STAT3 mutation type): SH2 – protein dimerization domain; DB – DNA-binding domain

Figure	Panels	Control cells used	Patients cells used
**Fig. 1**	**A, B**	CONTROL1; CONTROL2; CONTROL3; CONTROL4	AD-HIES1(SH2); AD-HIES2(DB); AD-HIES3(SH2); AD-HIES4(SH2); AD-HIES5(DB); AD-HIES6(DB); AD-HIES7(SH2)
	**C**	CONTROL1; CONTROL4; CONTROL3	AD-HIES3(SH2); AD-HIES4(SH2); AD-HIES6(DB)
**Fig. 2**	**C, D**		AD-HIES4(SH2); AD-HIES4(SH2)-STAT3 OVER
**Fig. 4**	**A**	CONTROL4 (shown)	AD-HIES4(SH2) (shown)
	**B**	CONTROL1; CONTROL4; CONTROL3	AD-HIES3(SH2); AD-HIES4(SH2); AD-HIES6(DB)
	**C**	CONTROL1; CONTROL2; CONTROL3; CONTROL4	AD-HIES1(SH2); AD-HIES2(DB); AD-HIES3(SH2); AD-HIES4(SH2); AD-HIES5(DB); AD-HIES6(DB); AD-HIES7(SH2)
**Figs 5, 6**		CONTROL1; CONTROL2; CONTROL3	AD-HIES3(SH2); AD-HIES4(SH2); AD-HIES6(DB)

Biology Open • Supplementary information

Table S2 (related to Fig. 6). Primer sequences that were used to check binding of STAT3 to promoters of NANOG and NANOGP8 genes in ChIP experiments shown on Fig. 6.

Gene promoter	Forward	Reverse
NANOG (Site 1)	GGGTTTGGGAATAGGAAGGAAAATC	GGAAGGGAGGTGTTTTCCAAAG
NANOG (Site 2)	CCATTCCTGTTGAACCATATTCCTG	AAACCGAGCAACAGAACCTGA
NANOG P8 (Site 1)	AAGATAAGCCAAAGGCCCTGGA	TGGTTCAGCGTAGGAACAGAAG
NANOG P8 (Site 2)	TGGGAACTCATGAAATGAATGCAAA	AAAGCTACCAAGGCTTTCTCCAG

Table S3. Primer sequences that were used for qPCR

Gene	Forward	Reverse
NANOG	AGGGAAACAACCCACTTCT	CCTTCTGCGTCACACCATT
STAT3	CAGCAGCTTGACACACGGTA	GCCCAATCTTGACTCTCAATCC

Biology Open • Supplementary information
